# Surviving in the cold: yeast mutants with extended hibernating lifespan are oxidant sensitive

**DOI:** 10.18632/aging.100104

**Published:** 2009-12-04

**Authors:** Lucie Postma, Hans Lehrach, Markus Ralser

**Affiliations:** ^1^ Max Planck Institute for Molecular Genetics, 14195 Berlin, Germany; ^2^ VU Medical Center Amsterdam, Dept. of Clinical Chemistry, 1081 HV Amsterdam, The Netherlands

**Keywords:** aging, metabolism, growth rate, oxidative stress resistance

## Abstract

Metabolic activity generates oxidizing molecules throughout life, but it is still debated if the resulting damage of macromolecules is a causality, or consequence, of the aging process. This problem demands for studying growth- and longevity phenotypes separately. Here, we assayed a complete collection of haploid Saccharomyces cerevisiae knock-out strains for their capacity to endure long periods at low metabolic rates. Deletion of 93 genes, predominantly factors of primary metabolism, allowed yeast to survive for more than 58 months in the cold. The majority of these deletion strains were not resistant against oxidants or reductants, but many were hypersensitive. Hence, survival at low metabolic rates has limiting genetic components, and correlates with stress resistance inversely. Indeed, maintaining the energy consuming anti-oxidative machinery seems to be disadvantageous under coldroom conditions.

Calorie restriction (CR), the practice of limiting caloric intake, retards aging phenotypes across species [1]. Furthermore, systematic exploration of the chronolo-gical (survival in the stationary phase) [2] and replicative (number of mitoses per mother) [3] lifespan of *S. cerevisiae* identified several metabolic genes and CR targets, such as the TOR pathway members, which lower metabolic activity and cause yeast lifespan extension when deleted. High metabolic turnover is a major source of free radicals and oxidative damage, other important players in the aging process. Many long-living mutations confer resistance against oxidants, and oxidatively damaged macromolecules are not inherited to yeast daughters [4]. There are profound observations that support the free radicals theory of aging. For instance, a recently identified yeast strain lacking *AFO1* is deficient in mitochondrial respiration, produces low amounts of free radicals and exhibits a massive lifespan extension of + 60% in median- and + 71% in maximum replicative lifespan [5].  However, despite these intense investigations, it is still unclear if the oxidative damage is indeed a cause, or simply a consequence, of the aging process itself [6]. A primary argument for the latter is the fact that genetic manipulations increasing the antioxidative capacity do generally not increase lifespan, in fact, many oxidant-resistant mutants are short living [7,8].

Hence, it would be important to generate data which allows distinguishing between growth rate, and long time survival. We speculated that identifying genetic factors which limit survival under conditions, at which the metabolic rate is naturally low, could bring us a step forward in solving this question.

Yeast kept at cold temperatures has a massively reduced growth- and metabolic rates; at 10°C the chronological lifespan is prolonged [9]. We arrayed a complete, S288c derived, *MAT*a knock-out collection onto 106 yeast peptone dextrose (YPD) petridishes. The plates were incubated at 30°C until giant colonies were formed, sealed and stored light protected in a cold room at 4°C. For assaying colony survival, plates were replicated onto fresh media and incubated at 30°C. After 12 months, most spots were still forming new colonies (Figure 1A). Thus, compared to higher temperature, yeast colonies kept at 4°C survive dramatically longer. Next, viability was assayed after an incubation time of 58 months. Now, most strains had lost their colony forming capacity. However a small fraction (2.3%) was still alive and produced giant colonies within 2 days after replication. These strains were exposed to a rigorous quality control and tested for methionine auxotrophy, kanamycin resistance and colour shifts upon CuSO_4_ treatment. Suspicious colonies were further analyzed be determination of mating capacity and auxotrophic markers. Finally, we verified the identity of all strains by amplifying and sequencing genetic barcodes.  Ultimately,  93 gene  deletions were confirmed; long-time survival in the cold is obviously limited by genetic components (Supplementary Table 1). To pay a tribute to mammals which can endure long winter periods at low metabolic rates, we propose the term *hibernating lifespan* for this yeast phenotype.

**Figure 1.**
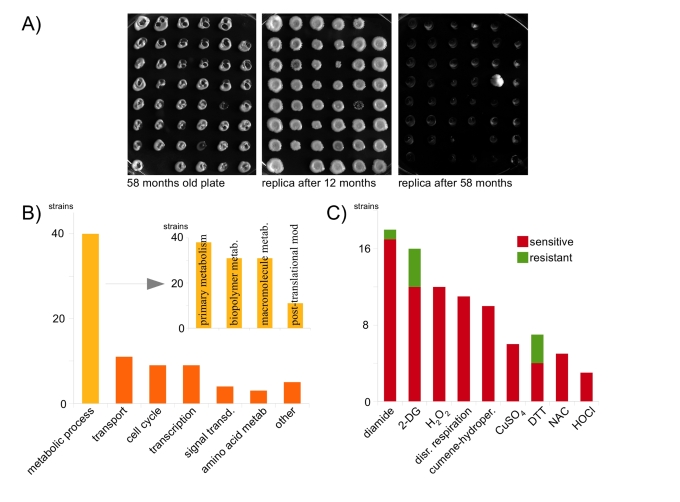
Oxidant-resistance is not a premise for long-time survival in the cold. (**A**) 106 48-position agar plates containing a systematic yeast knock-out collection were incubated at 4°C and replicated after 12 and 58 months, respectively. (**B**) GO analysis of surviving strains; the majority groups to metabolic processes (**C**) Comprehensive phenotypic analysis of mutants that survived for 58 months in the cold. Resistance to oxidants or reductants is the exception.

First, we compared these results with aging experiments performed at normal growth temperatures. No significant overlap with the systematic lifespan analysis [2,3,10] was observed, only one gene (*THI2*) exhibited a prolonged replicative lifespan. Thus, coldroom survival is neither a predictor for chronological, nor replicative aging. Most of the identified genes (43.5%) belong to the gene ontology (GO) term *metabolic process*, followed by *transport *(12%) and *cell cycle* (9.8%) (Figure 1B). *Metabolic process* genes were significantly enriched for terms *primary metabolism*,*biopolymer metabolism, macromolecular metabolism and post-translational protein modification *(P < 0.05).

To gain insights into the role of oxidant tolerances, we assayed the long-time survivors for potential phenotypes on multiple oxidants, reductants and related stressors (Supplementary Table 1, Figure 1C). Salt (NaCl) and polyamine (spermidine) tolerance was normal, and, compared to the wild-type, only three of the mutants were resistant, four sensitive, against the reductant dithiothreitol (DTT). Surprisingly, no strain was resistant to N-acetylcysteine (NAC), CuSO_4 _and hypochloric acid, some were sensitive (NAC: 5, CuSO_4_: 6_, _HOCl: 4). In addition, no strain was resistant against the classic oxidants H_2_O_2_ and cumol-hydroperoxide, only one (Δ*PUG1*) against diamide. Oxidant sensitivity, however, was common: 12 strains were sensitive to H_2_O_2_,10 to cumol-hydroperoxide and 17 to diamide. We further assayed the strains for potential deficits in mitochondrial activity, since the respiratory chain is a primary source for the production of free radicals under high metabolic rates. In agreement to the oxidant phenotype, no strain was deficient for respiration; all grew on non-fermentable carbon sources. However, for a quite significant number of mutants [11], mitochondrial respiration was essential: they were unable to grow after disruption of mitochondrial DNA by repeated ethidium bromide treatments. We wondered if this phenotype might correlate with resistance against the glycolytic inhibitor 2-deoxy-glucose (2-DG), whose toxicity increases with the rate of glycolysis or glucose uptake [11,12]. 16 strains showed a 2-DG phenotype, among these approx half of the strains for which respiration was essential, indicating that the primary energy metabolism was often affected in these mutants.

Thus, long-time survival at low temperatures has limiting genetic components that are, similar to mutations which retard ageing phenotypes, pre-dominately found among primary metabolic processes. However, oxidative stress resistance is not a premise for this phenotype. Indeed, the random occurrence of oxidant-sensitivity is much lower in the yeast knock-out collection [13]. It is evident that at low metabolic rates, less free radicals are released by the respiratory chain. Consequently, a highly active anti-oxidative system is not required; down-shutting of this energy consuming system seems to be advantageous.

Does hibernating lifespan resemble a classic aging phenotype? Cycles of death and growth allows bacterial cultures to maintain viable cells for very long time, a phenotype termed GASP (growth advantage in stationary phase). However, the longest surviving cultures may be composed of individual cells that are replicatively short-living [14]. In yeast, chronological lifespan is determined by monitoring the survival of a stationary cultures over time [15].  Also here, chronological ageing does not predict replicative ageing of individual cells [16]. Nonetheless, important conserved mechanisms of ageing were identified and understood in these experiments [2,15-17]. Similarly, *hibernating lifespan *may not be regarded as classic ageing phenotype. However, the fact that this dataset resembles a chronological ageing experiment performed at a very low temperature, it will be highly valuable in defining the role and consequence of free radicals and oxidative stress during ageing.

## Supplementary material

Supplementary Table 1 Gene deletion strains with extended hibernating lifespan and their phenotypes.
