# Supplementary material for: Surviving in the cold: yeast mutants with extended hibernating lifespan are oxidant sensitive
Source: Aging (Albany NY). 2009 Dec 4;1(11):957–60. doi: 10.18632/aging.100104 (PMC2815748; doi:10.18632/aging.100104)
Supplement: Supplementary Table 1 [file aging-01-957-s001.doc]

| Table S1 | |  | |  | | |  | |  | |  | |  | |  | Legend |  | |  | |  | |  |  |  |  |  |  |
| --- | --- | --- | --- | --- | --- | --- | --- | --- | --- | --- | --- | --- | --- | --- | --- | --- | --- | --- | --- | --- | --- | --- | --- | --- | --- | --- | --- | --- |
| **Yeast gene deletions which extend longtime survival in the cold and their phenotypes** | | | | | | | | | | | | | | | |  | resistant in comparison to wild-type | | | | |  | |  |  |  |  |  |
| Postma, Lehrach and Ralser | | | | | |  | |  | |  | |  | |  | |  | sensitive in comparison to wild-type | | | | |  | |  |  |  |  |  |
|  |  | |  | |  |  | |  | |  | |  | |  | |  |  |  | |  | |  | |  |  |  |  |  |
| **GeneName** | **AccNo** | | **SGD ID** | |  | **N-acetyl- cysteine** | | **CuSO4** | | **Spermidine** | | **HOCl** | | **DTT** | | **NaCl** | **H_2_O_2_** | **diamide** | | **Cumol**  **hydro**  **peroxide** | | **0.1% Glucose** | | **2% Glycerol** | **2% Galactose** | **Ethidium**  **Bromide** | **Growth after**  **2X Ethidium**  **Bromide** | **2-Deoxy- D-glucose** |
| **tested concentration range** | | |  | |  | **25 – 50 mM** | | **1.0 – 2.5 mM** | | **5-100mM** | | **0.01 - 0.05%** | | **2 – 3 mM** | | **3M** | **2.5 - 4 mM** | **1.2 – 1.8 mM** | | **0.1 – 0.15 mM** | |  | |  |  | **50 ng/ml** | **2* 50 ng/ml** | **0.1 – 0.25%** |
| YAL036C | AAC04995.1 | | S000000034 | | U12980 |  | |  | |  | |  | |  | |  |  |  | |  | |  | |  |  |  |  |  |
| YAR040C | AAC04995.1 | | S000000034 | | U12980 |  | |  | |  | |  | |  | |  |  |  | |  | |  | |  |  | **DeepRed colour** |  |  |
| YBL056W | CAA80791.1 | | S000000152 | | Z23261 |  | |  | |  | |  | |  | |  |  |  | |  | |  | |  |  | **DeepRed colour** |  |  |
| YBR048W | AAC37410.1 | | S000000252 | | L17004 |  | |  | |  | |  | |  | |  |  |  | |  | |  | |  |  |  |  |  |
| YBR073W | CAA85017.1 | | S000000277 | | Z35942 |  | |  | |  | |  | |  | |  |  |  | |  | |  | |  |  |  |  |  |
| YBR082C | CAA35529.1 | | S000000286 | | X17494 |  | |  | |  | |  | |  | |  |  |  | |  | |  | |  |  |  |  |  |
| YBR114W | AAA34931.1 | | S000000318 | | M86929 |  | |  | |  | |  | |  | |  |  |  | |  | |  | |  |  |  |  |  |
| YBR129C | CAA85086.1 | | S000000333 | | Z35998 |  | |  | |  | |  | |  | |  |  |  | |  | |  | |  |  |  |  |  |
| YBR137W | BAA01861.1 | | S000000341 | | D11088 |  | |  | |  | |  | |  | |  |  |  | |  | |  | |  |  |  |  |  |
| YBR141C | CAA55538.1 | | S000000345 | | X78937 |  | |  | |  | |  | |  | |  |  |  | |  | |  | |  |  |  |  |  |
| YBR146W | CAA85104.1 | | S000000350 | | Z36015 |  | |  | |  | |  | |  | |  |  |  | |  | |  | |  |  |  |  |  |
| YBR148W | CAA85106.1 | | S000000352 | | Z36017 |  | |  | |  | |  | |  | |  |  |  | |  | |  | |  |  |  |  |  |
| YBR151W | CAA85109.1 | | S000000355 | | Z36020 |  | |  | |  | |  | |  | |  |  |  | |  | |  | |  |  | **DeepRed colour** |  |  |
| YBR240C | BAA22093.1 | | S000000444 | | D32073 |  | |  | |  | |  | |  | |  |  |  | |  | |  | |  |  |  |  |  |
| YBR300C | CAA85265.1 | | S000000504 | | Z36169 |  | |  | |  | |  | |  | |  |  |  | |  | |  | |  |  |  |  |  |
| YCL042W | CAC42956.1 | | S000000547 | | X59720 |  | |  | |  | |  | |  | |  |  |  | |  | |  | |  |  |  |  |  |
| YCR021C | AAA02903.1 | | S000000615 | | M93123 |  | |  | |  | |  | |  | |  |  |  | |  | |  | |  |  |  |  |  |
| YDL056W | CAA98618.1 | | S000002214 | | Z74104 |  | |  | |  | |  | |  | |  |  |  | |  | |  | |  |  |  |  |  |
| YDL136W | AAA35000.1 | | S000002295 | | L02328 |  | |  | |  | |  | |  | |  |  |  | |  | |  | |  |  |  |  |  |
| YDR104C | CAA87680.1 | | S000002511 | | Z47746 |  | |  | |  | |  | |  | |  |  |  | |  | |  | |  |  | **DeepRed colour** |  |  |
| YDR121W | CAA88674.1 | | S000002528 | | Z48758 |  | |  | |  | |  | |  | |  |  |  | |  | |  | |  |  |  |  |  |
| YDR139C | CAA76516.1 | | S000002546 | | Y16890 |  | |  | |  | |  | |  | |  |  |  | |  | |  | |  |  |  |  |  |
| YDR144C | AAC49112.1 | | S000002551 | | U14733 |  | |  | |  | |  | |  | |  |  |  | |  | |  | |  |  |  |  |  |
| YDR216W | AAA73863.1 | | S000002624 | | U28414 |  | |  | |  | |  | |  | |  |  |  | |  | |  | |  |  |  |  |  |
| YDR403W | AAB64843.1 | | S000002811 | | U32274 |  | |  | |  | |  | |  | |  |  |  | |  | |  | |  |  |  |  |  |
| YDR428C | AAB64885.1 | | S000002836 | | U33007 |  | |  | |  | |  | |  | |  |  |  | |  | |  | |  |  |  |  |  |
| YDR438W | AAB64860.1 | | S000002846 | | U33007 |  | |  | |  | |  | |  | |  |  |  | |  | |  | |  |  | **DeepRed colour** |  |  |
| YEL004W | AAB64481.1 | | S000000730 | | U18530 |  | | **no colour shift, *MAT*a confirmed** | |  | |  | |  | |  |  |  | |  | |  | |  |  |  |  |  |
| YEL017C-A |  | | S000002103 | |  |  | |  | |  | |  | |  | |  |  |  | |  | |  | |  |  |  |  |  |
| YER185W | AAB64712.1 | | S000000987 | | U18922 |  | |  | |  | |  | |  | |  |  |  | |  | |  | |  |  |  |  |  |
| YFL035C-B |  | | S000001859 | |  |  | |  | |  | |  | |  | |  |  |  | |  | |  | |  |  |  |  |  |
| YFR025C | AAA18399.1 | | S000001921 | | U09479 |  | |  | |  | |  | |  | |  |  |  | |  | |  | |  |  |  |  |  |
| YGL021W | CAA96721.1 | | S000002989 | | Z72543 |  | |  | |  | |  | |  | |  |  |  | |  | |  | |  |  |  |  |  |
| YGL037C | CAA96739.1 | | S000003005 | | Z72559 |  | |  | |  | |  | |  | |  |  |  | |  | |  | |  |  |  |  |  |
| YGL042C | CAA96745.1 | | S000003010 | | Z72565 |  | |  | |  | |  | |  | |  |  |  | |  | |  | |  |  |  |  |  |
| YGL165C | CAA96878.1 | | S000003133 | | Z72688 |  | |  | |  | |  | |  | |  |  |  | |  | |  | |  |  |  |  |  |
| YGR031W | CAA97019.1 | | S000003263 | | Z72816 |  | |  | |  | |  | |  | |  |  |  | |  | |  | |  |  |  |  |  |
| YGR122W | CAA97133.1 | | S000003354 | | Z72907 |  | |  | |  | |  | |  | |  |  |  | |  | |  | |  |  |  |  |  |
| YGR154C | CAA97168.1 | | S000003386 | | Z72939 |  | |  | |  | |  | |  | |  |  |  | |  | |  | |  |  |  |  |  |
| YGR182C | CAA67527.1 | | S000003414 | | X99074 |  | |  | |  | |  | |  | |  |  |  | |  | |  | |  |  |  |  |  |
| YGR189C | CAA67525.1 | | S000003421 | | X99074 |  | |  | |  | |  | |  | |  |  |  | |  | |  | |  |  |  |  |  |
| YGR209C | AAA85584.1 | | S000003441 | | U40843 |  | |  | |  | |  | |  | |  |  |  | |  | |  | |  |  |  |  |  |
| YGR259C | CAA97288.1 | | S000003491 | | Z73044 |  | |  | |  | |  | |  | |  |  |  | |  | |  | |  |  |  |  |  |
| YGR260W | CAA97289.1 | | S000003492 | | Z73044 |  | |  | |  | |  | |  | |  |  |  | |  | |  | |  |  |  |  |  |
| YHR005C | AAB68432.1 | | S000001047 | | U10555 |  | |  | |  | |  | |  | |  |  |  | |  | |  | |  |  |  |  |  |
| YHR079C | AAA34489.1 | | S000001121 | | L19640 |  | |  | |  | |  | |  | |  |  |  | |  | |  | |  |  |  |  |  |
| YHR094C | AAB59311.1 | | S000001136 | | L07079 |  | |  | |  | |  | |  | |  |  |  | |  | |  | |  |  |  |  |  |
| YHR199C | AAB68371.1 | | S000001242 | | U00030 |  | |  | |  | |  | |  | |  |  |  | |  | |  | |  |  |  |  |  |
| YIL076W | CAA86094.1 | | S000001338 | | Z37997 |  | |  | |  | |  | |  | |  |  |  | |  | |  | |  |  | **DeepRed colour** |  |  |
| YIL100W | CAA86281.1 | | S000001362 | | Z38125 |  | |  | |  | |  | |  | |  |  |  | |  | |  | |  |  |  |  |  |
| YIL112W | CAA86268.1 | | S000001374 | | Z38125 |  | |  | |  | |  | |  | |  |  |  | |  | |  | |  |  |  |  |  |
| YJL169W | CAA89463.1 | | S000003705 | | Z49444 |  | |  | |  | |  | |  | |  |  |  | |  | |  | |  |  |  |  |  |
| YJR107W | CAA89637.1 | | S000003868 | | Z49607 |  | |  | |  | |  | |  | |  |  |  | |  | |  | |  |  |  |  |  |
| YJR125C | CAA89656.1 | | S000003886 | | Z49625 |  | |  | |  | |  | |  | |  |  |  | |  | |  | |  |  |  |  |  |
| YKL075C | CAA81913.1 | | S000001558 | | Z28075 |  | |  | |  | |  | |  | |  |  |  | |  | |  | |  |  |  |  |  |
| YKL092C | CAA81930.1 | | S000001575 | | Z28092 |  | |  | |  | |  | |  | |  |  |  | |  | |  | |  |  |  |  |  |
| YKL142W | CAA81983.1 | | S000001625 | | Z28142 |  | |  | |  | |  | |  | |  |  |  | |  | |  | |  |  |  |  |  |
| YKL151C | CAA81992.1 | | S000001634 | | Z28151 |  | |  | |  | |  | |  | |  |  |  | |  | |  | |  |  |  |  |  |
| YKR007W | CAA82077.1 | | S000001715 | | Z28232 |  | |  | |  | |  | |  | |  |  |  | |  | |  | |  |  |  |  |  |
| YLL054C | CAA97507.1 | | S000003977 | | Z73159 |  | |  | |  | |  | |  | |  |  |  | |  | |  | |  |  |  |  |  |
| YLL056C | CAA97509.1 | | S000003979 | | Z73161 |  | |  | |  | |  | |  | |  |  |  | |  | |  | |  |  |  |  |  |
| YLR024C | CAA97547.1 | | S000004014 | | Z73196 |  | |  | |  | |  | |  | |  |  |  | |  | |  | |  |  |  |  |  |
| YLR068W | CAA97624.1 | | S000004058 | | Z73240 |  | |  | |  | |  | |  | |  |  |  | |  | |  | |  |  |  |  |  |
| YLR095C | AAB67539.1 | | S000004085 | | U53876 |  | |  | |  | |  | |  | |  |  |  | |  | |  | |  |  |  |  |  |
| YLR455W | AAB64719.1 | | S000004447 | | U22383 |  | |  | |  | |  | |  | |  |  |  | |  | |  | |  |  |  |  |  |
| YML064C | CAA86257.1 | | S000004529 | | Z38114 |  | |  | |  | |  | |  | |  |  |  | |  | |  | |  |  | **DeepRed colour** |  |  |
| YMR073C | CAA88798.1 | | S000004677 | | Z48952 |  | |  | |  | |  | |  | |  |  |  | |  | |  | |  |  |  |  |  |
| YMR075C-A | CAA88798.1 | | S000004677 | | Z48952 |  | |  | |  | |  | |  | |  |  |  | |  | |  | |  |  |  |  |  |
| YMR075W | CAA88800.1 | | S000004680 | | Z48952 |  | |  | |  | |  | |  | |  |  |  | |  | |  | |  |  |  |  |  |
| YMR289W | CAA88800.1 | | S000004680 | | Z48952 |  | |  | |  | |  | |  | |  |  |  | |  | |  | |  |  |  |  |  |
| YMR316C-A | CAA88800.1 | | S000004680 | | Z48952 |  | |  | |  | |  | |  | |  |  |  | |  | |  | |  |  |  |  |  |
| YNL034W | CAA95880.1 | | S000004979 | | Z71294 |  | |  | |  | |  | |  | |  |  |  | |  | |  | |  |  |  |  |  |
| YNR001C | CAA25359.1 | | S000005284 | | X00782 |  | |  | |  | |  | |  | |  |  |  | |  | |  | |  |  |  |  |  |
| YNR028W | CAA96308.1 | | S000005311 | | Z71643 |  | |  | |  | |  | |  | |  |  |  | |  | |  | |  |  |  |  |  |
| YOR040W | CAA99230.1 | | S000005566 | | Z74948 |  | |  | |  | |  | |  | |  |  |  | |  | |  | |  |  |  |  |  |
| YOR088W | CAA99230.1 | | S000005566 | | Z74948 |  | |  | |  | |  | |  | |  |  |  | |  | |  | |  |  |  |  |  |
| YOR093C | CAA99290.1 | | S000005619 | | Z75001 |  | |  | |  | |  | |  | |  |  |  | |  | |  | |  |  | **DeepRed colour** |  |  |
| YOR138C | CAA99337.1 | | S000005664 | | Z75046 |  | |  | |  | |  | |  | |  |  |  | |  | |  | |  |  |  |  |  |
| YOR142W | CAA64059.1 | | S000005668 | | X94335 |  | |  | |  | |  | |  | |  |  |  | |  | |  | |  |  |  |  |  |
| YOR229W | CAA99449.1 | | S000005755 | | Z75137 |  | |  | |  | |  | |  | |  |  |  | |  | |  | |  |  |  |  |  |
| YOR240W | CAA99449.1 | | S000005755 | | Z75137 |  | |  | |  | |  | |  | |  |  |  | |  | |  | |  |  | **DeepRed colour** |  |  |
| YOR327C | CAA99647.1 | | S000005854 | | Z75235 |  | |  | |  | |  | |  | |  |  |  | |  | |  | |  |  |  |  |  |
| YPL034W | AAB68185.1 | | S000005955 | | U44030 |  | |  | |  | |  | |  | |  |  |  | |  | |  | |  |  |  |  |  |
| YPL108W | AAB68192.1 | | S000006029 | | U43281 |  | |  | |  | |  | |  | |  |  |  | |  | |  | |  |  |  |  |  |
| YPL114W | AAB68253.1 | | S000006035 | | U43503 |  | |  | |  | |  | |  | |  |  |  | |  | |  | |  |  |  |  |  |
| YPL170W | CAA97876.1 | | S000006091 | | Z73526 |  | |  | |  | |  | |  | |  |  |  | |  | |  | |  |  |  |  |  |
| YPL177C | CAA97884.1 | | S000006098 | | Z73533 |  | |  | |  | |  | |  | |  |  |  | |  | |  | |  |  |  |  |  |
| YPL192C | CAA97905.1 | | S000006113 | | Z73548 |  | |  | |  | |  | |  | |  |  |  | |  | |  | |  |  |  |  |  |
| YPL221W | CAA97936.1 | | S000006142 | | Z73577 |  | |  | |  | |  | |  | |  |  |  | |  | |  | |  |  |  |  |  |
| YPR007C | CAA88785.1 | | S000006211 | | Z48951 |  | |  | |  | |  | |  | |  |  |  | |  | |  | |  |  |  |  |  |
| YPR023C | CAA89277.1 | | S000006227 | | Z49274 |  | |  | |  | |  | |  | |  |  |  | |  | |  | |  |  |  |  |  |
| YPR125W | AAB68065.1 | | S000006329 | | U40828 |  | |  | |  | |  | |  | |  |  |  | |  | |  | |  |  |  |  |  |
|  |  | |  | | **total count sensitive** | **5** | | **6** | | No difference to WT up to 100mM | | **3** | | **4** | | No difference to WT up to 3M | **12** | **17** | | **10** | | No phenotypes | | No phenotypes | No phenotypes | 9 acquired a deep red colour | **11** | **12** |
|  |  | |  | | **total count resistant** | **0** | | **0** | |  | | **0** | | **3** | |  | **0** | **1** | | **0** | |  | |  |  |  | **n.a.** | **4** |
